# Comparison of methods for determining the effectiveness of antibacterial functionalized textiles

**DOI:** 10.1371/journal.pone.0188304

**Published:** 2017-11-21

**Authors:** Hajo Haase, Lisa Jordan, Laura Keitel, Claudia Keil, Boris Mahltig

**Affiliations:** 1 Department of Food Chemistry and Toxicology, Institute for Food Technology and Food Chemistry, Technische Universität Berlin, Berlin, Germany; 2 Faculty of Textile and Clothing Technology, Hochschule Niederrhein, University of Applied Science, Mönchengladbach, Germany; Institute of Materials Science, GERMANY

## Abstract

Antimicrobial functionalization of textiles is important for various applications, such as protection of textile materials from decomposition, generation of more effective wound dressings, and the prevention of infections or malodors resulting from bacterial growth. In order to test the efficacy of new products, their antibacterial activity needs to be evaluated. At present, several different procedures are being used for this purpose, hindering comparisons among different studies. The present paper compares five of these assays using a sample panel of different textiles functionalized with copper (Cu) and silver (Ag) as antibacterial agents, and discusses the suitability of these methods for different analytical requirements. Bacterial viability was determined by measuring the optical density at 600 nm, a colorimetric assay based on MTT (3-[4, 5-dimethylthiazol-2-yl]-2, 5 diphenyl tetrazolium bromide) conversion, an agar diffusion assay, and colony formation, either after culturing in media containing textile samples, or after recovery from textiles soaked with bacterial suspension. All experiments were performed with a Gram-negative (*Escherichia coli*) and a Gram-positive (*Staphylococcus warneri*) model organism. In general, the results yielded by the different methods were of good comparability. To identify the most suitable test system for the particular type of antibacterial coating, several factors need to be taken into account, such as choosing appropriate endpoints for analyzing passive or active antibacterial effects, selection of relevant microorganisms, correcting for potential interference by leaching of colored textile coatings, required hands on time, and the necessary sensitivity.

## Introduction

Antimicrobial functionalization of textiles has multiple applications. It can be used to protect textiles from microbial growth and degradation, is used in medical applications such as wound dressings and to prevent secondary infections of the skin under conditions such as atopic dermatitis, and may be applied to prevent odor formation resulting from bacterial metabolites on clothes or textiles used for cleaning purposes [1–3]. To this end, textiles are treated with agents containing various substances with antibacterial properties. These include, amongst many others, antibiotics such as Ciprofloxacin, disinfectants such as Triclosan, polysaccharides such as chitosan, and metal-based antibacterial coatings, in particular ones containing silver, zinc or copper [1,4,5].

Antibacterial substances can either act actively, having a direct bactericidal effect or hindering growth without killing the bacteria, or they act passively by impeding bacteria to colonize the textile fibers, e.g., by obstructing bacterial adhesion [2]. To evaluate and compare the effectiveness of different textile treatments, their antibacterial activity needs to be experimentally confirmed, as there is considerable variation in the activity of different products [6]. At present, various different methods are being used for testing the effectiveness of the different coatings:

The colorimetric MTT assay is based on the reduction of the tetrazole 3-(4,5-dimethylthiazol-2-yl)-2,5-diphenyltetrazolium bromide (MTT) to its respective formazan. The resulting purple dye is then quantified at 570 nm in a photometer or multiplate reader. This test is commonly used for measuring proliferation and viability of mammalian cells, but MTT and other related substances, such as 2,3,5-triphenyl-tetrazolium chloride, can also be utilized for measuring bacterial viability [7]. In fact, the use of MTT for testing bactericidal activity of functionalized textiles has already been reported several times [8–10].

The disk diffusion method had initially been applied for testing the efficacy of antibiotics [11]. Bacteria are incubated on an agar plate on which a filter is placed, which has been loaded with the test compound(s). Antibacterial activity is then determined by the absence of bacterial growth in a zone around the filter. Modified versions of this method have been used for testing the toxicity of nanoparticle solutions in a well in the agar [12], or by substituting the filter for a textile sample, so that its antimicrobial effectiveness can be evaluated in a comparable manner [8,13,14].

Another method to quantify bacterial growth is by measuring the optical density at 600 nm (OD_600_), which is based on turbidity resulting from light scattering by bacteria [15]. This is mainly used as a quick and affordable method to monitor growth of bacteria during their culture in liquid media, but has also been applied for testing antibacterial properties of nanostructures [7,16], and might be a suitable method for evaluating antibacterial effects of textile samples, as well.

Counting the number of viable bacteria by formation of visible colonies on agar plates, known as colony formation, is another method for determining bacterial viability. It can either be performed using bacterial solutions in which textile samples had been incubated, or on bacteria that were eluted from a bacteria-soaked textile sample. The latter is the basic principle underlying the protocol 100–2004 recommended by the American Association of Textile Chemists and Colorists (AATCC) to assess the efficacy of antibacterial finishes, and of the corresponding norm EN ISO 20743 by the International Organization for Standardization. Both protocols, or various modifications thereof, are frequently used for investigating antibacterial properties of fabrics [17–21].

One important obstacle that is currently hindering the development of effective antibacterial textiles is the lack of comparability between different studies [22]. So far, only few publications use more than one method to assess antibacterial activity, leading to extremely limited information on potential differences between the results of commonly used test methods. Therefore, the present study uses a sample set of antibacterial furnished textiles, based on copper or silver as active ingredients, aiming to evaluate the comparability of the results obtained with five different test methods and to discuss the methods’ suitability for evaluating the antibacterial properties of textiles.

## Materials and methods

### Textile samples

A panel of 28 different textile samples was prepared following a coating procedure already described in detail [23]. Briefly, a polyester filament fabric with a specific weight of 180 g/cm^2^ was used as a substrate. It was coated with layers of 50 μm, 100 μm or 200 μm thickness, consisting of acrylate or acrylate/polyaniline coatings, which were supplemented with up to 30% copper or silver pigments. For the present experiments, circles with 5 mm diameter were prepared using a conventional hole puncher.

### Bacterial culture

*E*. *coli* (strain BL21(D3)) were cultured in Luria–Bertani (LB) medium. *S*. *warneri* (strain dsm-20316) were grown in Trypticase Soy Yeast Extract (TYSE) medium. Glycerol stocks were expanded in overnight cultures at 37°C and 150 rpm. For the experiments, cells were diluted 1:250 in their respective culture media and treated as described below. A growth curve indicates that both bacterial strains are in a phase of logarithmic growth after 3h (S1 Fig). Consequently, this has been used as the incubation time for subsequent experiments.

### MTT assay

Antimicrobial testing based on the reduction of MTT was done as described elsewhere [8]. Briefly, 200 μl bacterial suspension per cavity were grown in sterile 96-multiwell cell culture plates from TPP (Techno Plastic Products AG, Trasadingen, Switzerland) in their respective culture media and in the presence of textile samples for 3h at 37°C, rotating at 250 rpm in a PST-60-HL-4 orbital shaker (BioSan, Riga, Latvia). Subsequently, cells were incubated in the presence of 0.1 mg/l MTT in culture medium for 5 min, followed by lysis in isopropanol for further 30 min and determination of the absorption at 570 nm with a reference wavelength of 630 nm on an ELx800 absorbance microplate reader (BioTek Instruments, Winooski, USA). The control viability of 100% was measured in a test arrangement in the absence of any textile fabric, but otherwise identical to the other samples; 0% was determined in the same setup in the absence of bacteria.

### OD_600_ assay

Cells were grown in 96 microwell plates in the presence of textile disks as described for the MTT assay. After the incubations, 100 μl of the bacterial suspensions were transferred to a new plate and absorption was measured at 600 nm on an Infinite M200 microplate reader (Tecan, Crailsheim, Germany). 100% viability was defined as bacterial grown in the absence of textile samples, 0% viability as medium blanks.

### Disk diffusion assay

The disk diffusion assay was performed as previously described [8]. In brief, textile disks were placed on LB or TSYE agar plates on which bacterial suspensions had been seeded with a Drigalski spatula. After incubation for 24h at 37°C, a caliper gauge was used to determine the width of the bacteria-free areola. Results are given as the area of the zone of growth inhibition in mm^2^.

### Colony formation with incubation in solution (Colony^(Sol)^)

Cells were grown on 96 microwell plates in the presence of textile disks as described for the MTT assay. Afterwards a series of 10-fold dilutions was prepared, of which 10 μl aliquots were placed on LB or TSYE agar plates in triplicates and incubated for 24 h at 37°C. Colonies were counted for the first dilution at which distinguishable colonies were observable and used to calculate the number of viable bacteria in the original solution.

### Colony formation on soaked textile disks (Colony^(Soak)^)

Textile disks were soaked with 20 μl bacterial suspension and incubated for 3h at 37°C in a humidified atmosphere. Afterwards, bacteria were eluted in 100 μl 0.85% NaCl solution for 1 min. Serial dilution and colony formation were performed as described above. Data are shown as the percentage of viable cells recovered from the textile sample at 3h, relative to recovery from the respective sample at 0h. The quantitative retrieval of bacteria from the textiles was efficient; the average recovery from all textile samples immediately after soaking compared to the amount of bacteria originally placed onto the textiles was 86.4% for *E*. *coli* and 114.5% for *S*. *warneri*, respectively.

### Measuring metal ion release by atomic absorption spectrometry (AAS)

To analyze metal release, textile disks were placed into 200 μl culture media in 96 well plates and incubated for 3h at 37°C, rotating at 250 rpm. Afterwards, media were diluted in 0.65% HNO_3_ and analyzed by flame AAS on an AAnalyst 800 instrument (Perkin Elmer, Rodgau, Germany), using the conditions summarized in Table 1. Acetylene flow was set to 2.0 l/min and oxidant (air) to 17.0 l/min; all samples were analyzed in triplicates. Reagents for atomic absorption measurements were of appropriate quality for trace element analysis (TraceSelect, Fluka, Germany).

**Table 1:** Conditions for AAS.

Element	Wavelength [nm]	Slit Width [nm]	Dilution (v/v)	Standard curve [mg/l]
Ag	328.1	0.7	1:5	0.1–2.5
Cu	324.8	0.7	1:50	0.5–5

### Statistical analyses

All experiments were performed independently at least three times and results are shown as means with the standard error of means (S.E.M.) as error bars. Statistical significance of the experimental results (significance level of p<0.05) was calculated by GraphPad prism software (version 5.0, GraphPad Software, San Diego USA) using one-way ANOVA and Tukey´s post-hoc test. To compare the results obtained with the different test methods, data were analyzed by linear correlation analysis calculating the Pearson product-moment correlation coefficient.

## Results

**Fig 1:**
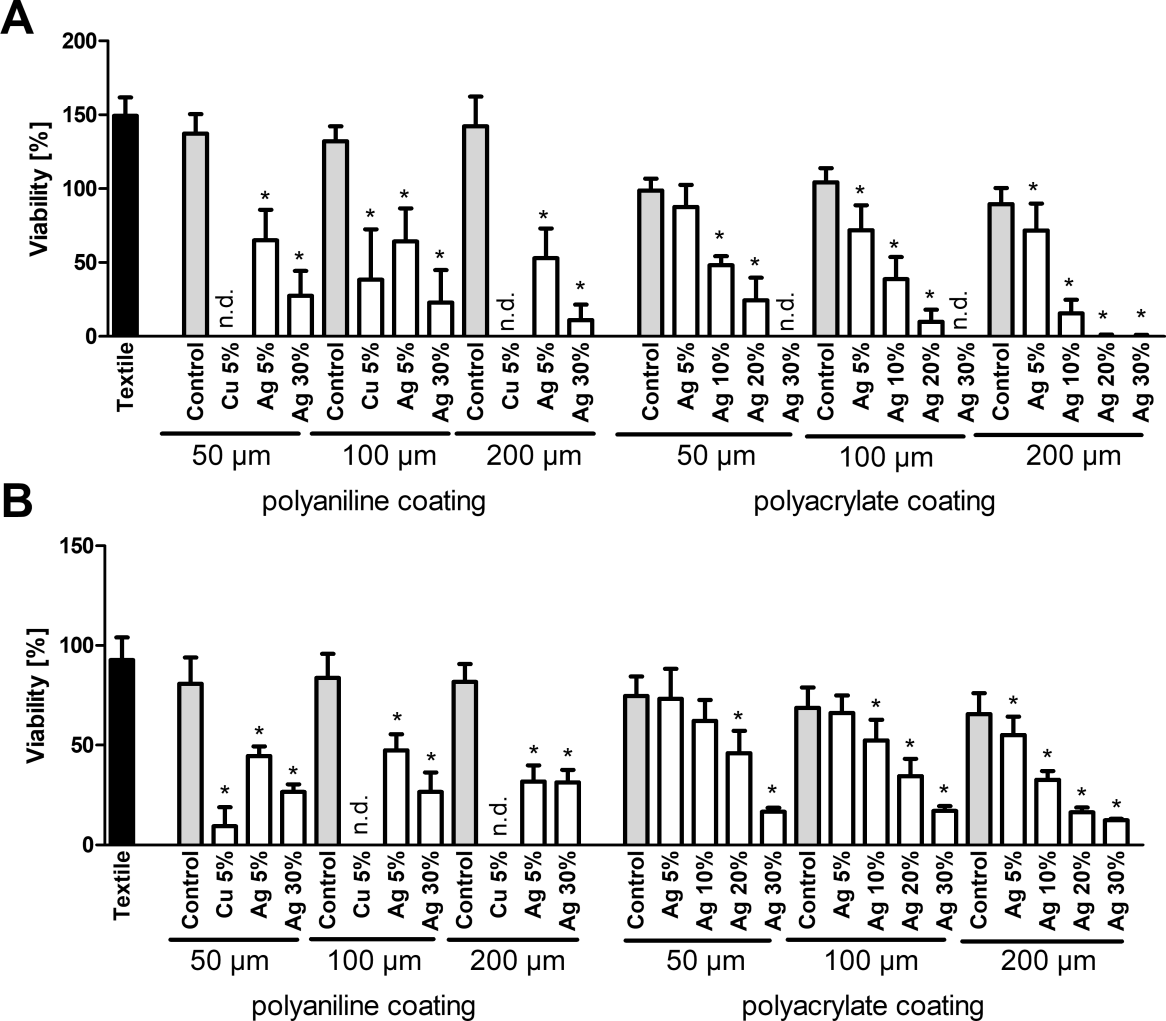
MTT assay. Viability of *E*. *coli* (A) or *S*. *warneri* (B) after 3h culture in the presence of textile samples was analyzed by the MTT assay. A viability of 100% corresponds to the signal obtained with bacteria in the absence of any textile samples. Data are shown as means +S.E.M. of n = 6 independent experiments. *Mean significantly different from untreated textile; One-way ANOVA with Tukey´s post-hoc test (p < 0.05). n.d.: not detected.

**Fig 2:**
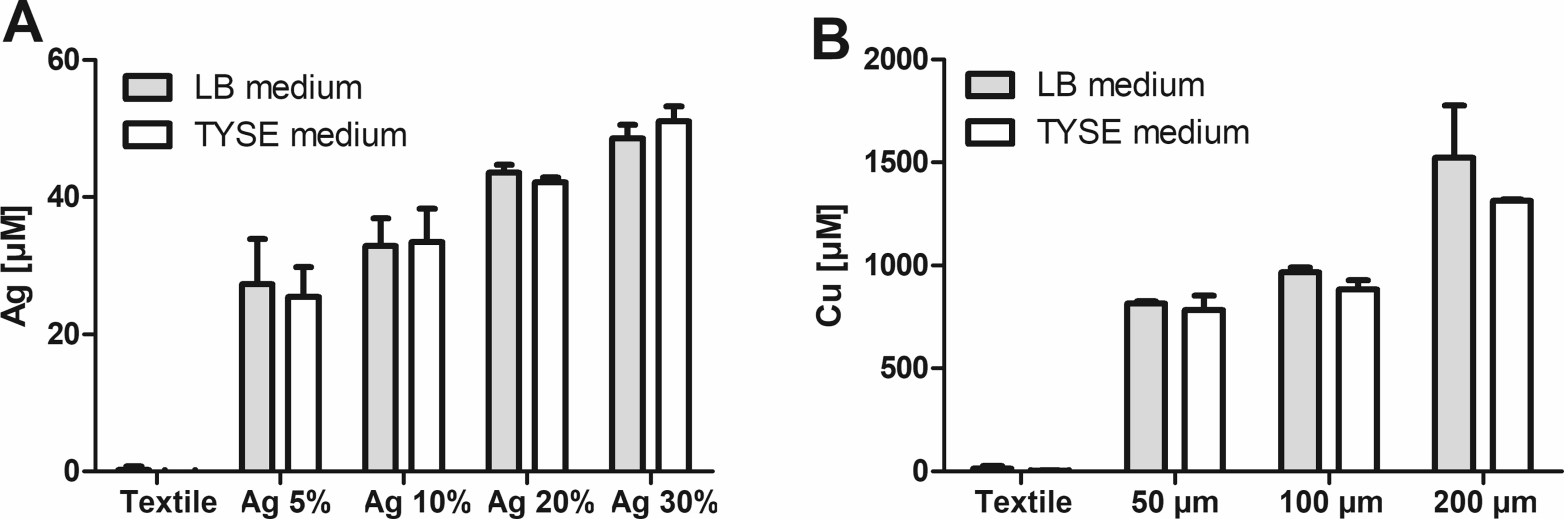
Metal release. To monitor metal release during incubation of the bacteria, textile samples were kept for 3h at 37°C in LB or TYSE media on a rotating incubator. Subsequently, the total amounts of Ag released from polyacrylate coating, thickness 200 μm (A) or Cu released from polyaniline coating containing 5% Cu (B) in the media were quantified by AAS. Data are shown as means +S.E.M. of n = 3 independent experiments.

An MTT assay of bacteria cultured in the presence of textile samples shows that addition of copper or silver pigments to textile coatings applied onto a polyester fabric reduces the viability of *E*. *coli* (Fig 1A) and *S*. *warneri* (Fig 1B). For silver, which has been applied in four different concentrations (ranging from 5 to 30%) in a polyacrylate coating, a concentration-dependent effect is observed. This correlates with a concentration-dependent silver release into the culture media, as shown in Fig 2A for the coating of 200 μm thickness. Analogous results are also observed for all other silver-containing coatings (data not shown). Remarkably, in the MTT assay textiles furnished with copper show higher toxicity than the corresponding silver-treated samples. In contrast, silver ions (Ag^+^) in solution are more toxic than copper ions (Cu^2+^), showing lower EC_50_ values for both types of bacteria (S2 Fig). These observations are reconciled by measurements of metal release into the culture media by AAS. The resulting concentrations of copper are millimolar, and hereby more than one order of magnitude higher than those of silver (Fig 2B).

**Fig 3:**
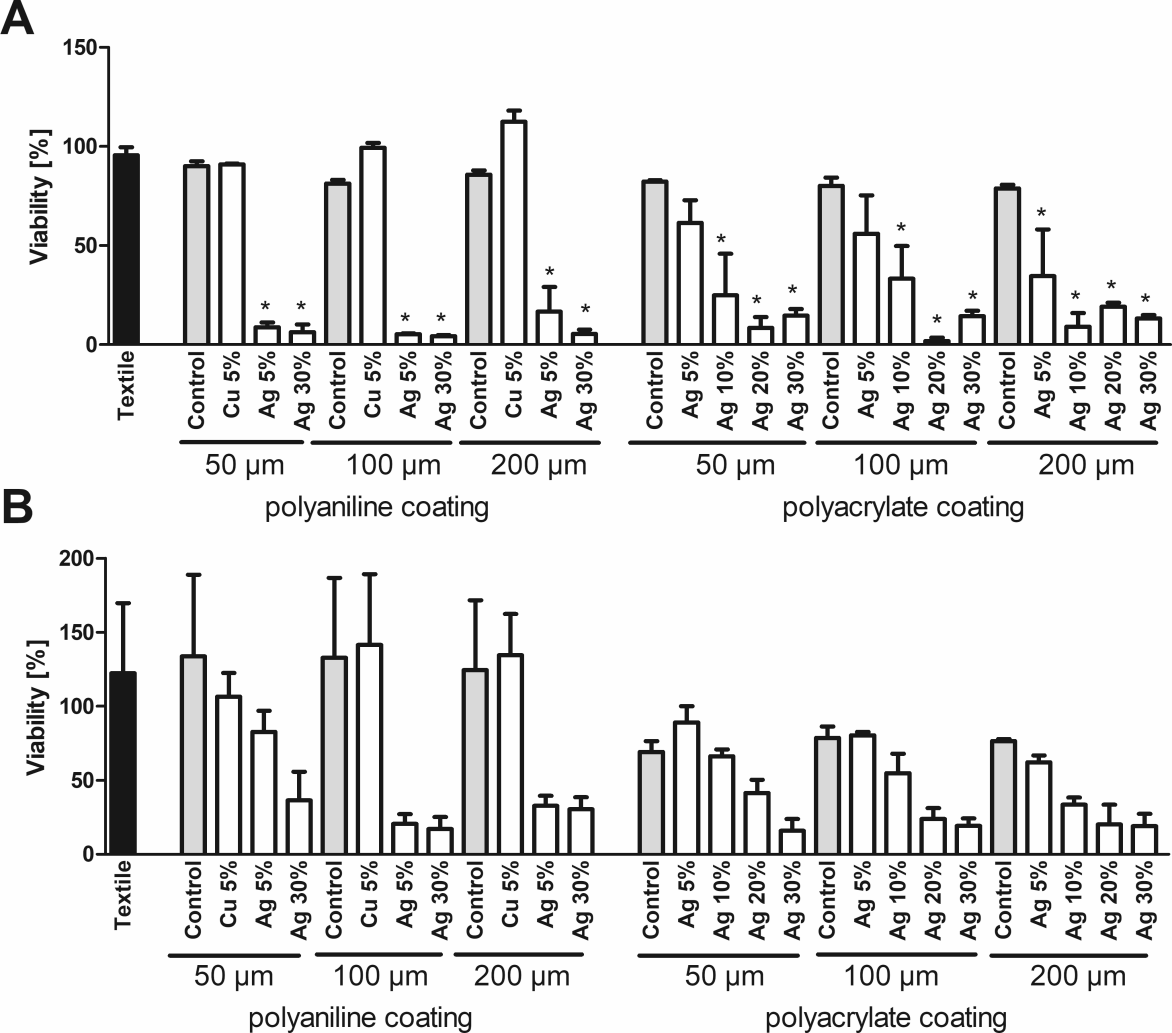
OD_600_ assay. Viability of *E*. *coli* (A) or *S*. *warneri* (B) after 3h culture in the presence of textile samples was analyzed by measuring the optical density at 600 nm. A viability of 100% corresponds to the signal obtained with bacteria in the absence of any textile samples. Data are shown as means +S.E.M. of n = 3 independent experiments. *Mean significantly different from untreated textile; One-way ANOVA with Tukey´s post-hoc test (p < 0.05).

**Fig 4:**
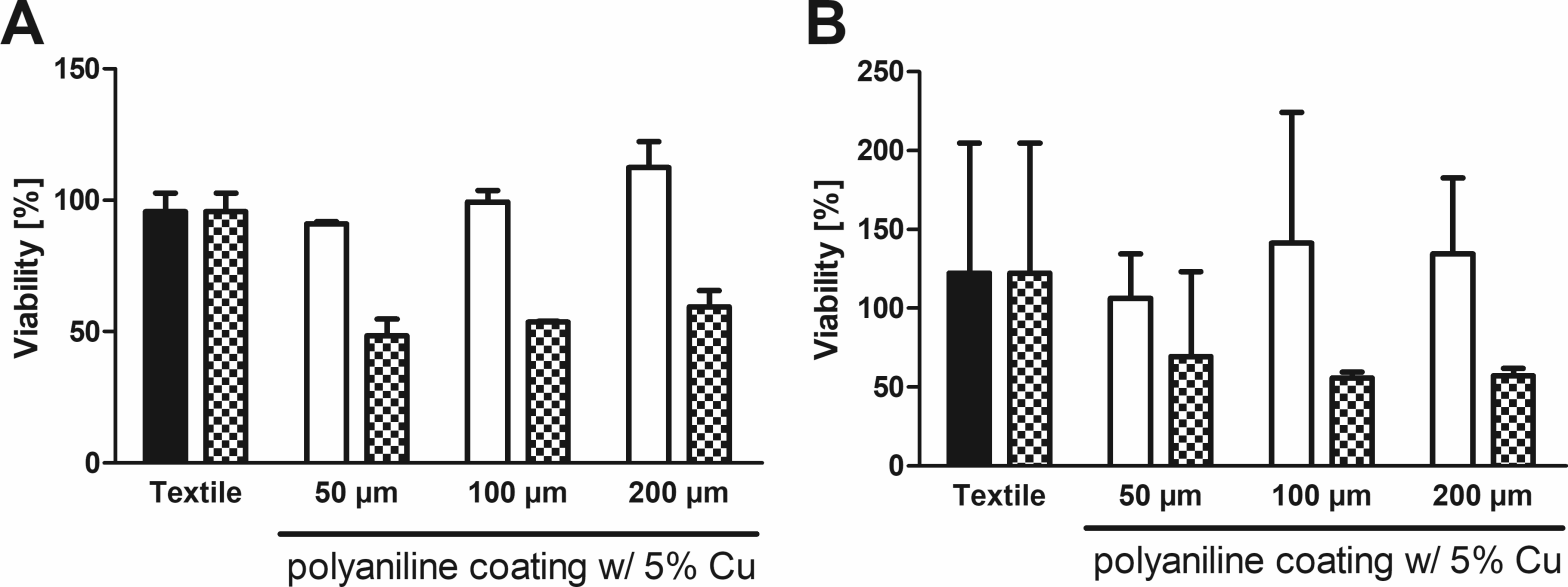
OD_600_ assay with background correction. In a complementary experiment to Fig 2, the effect of leaching of coating from the textile samples after incubation in bacteria-free media for *E*. *coli* (LB-medium, A) or *S*. *warneri* (TYSE-medium, B) was measured at 600 nm. Original data (monochromatic bars) and data after correction by subtracting the absorption at 600 nm caused by leaching (checkered bars) are shown as means +S.E.M. of n = 3 independent experiments.

**Fig 5:**
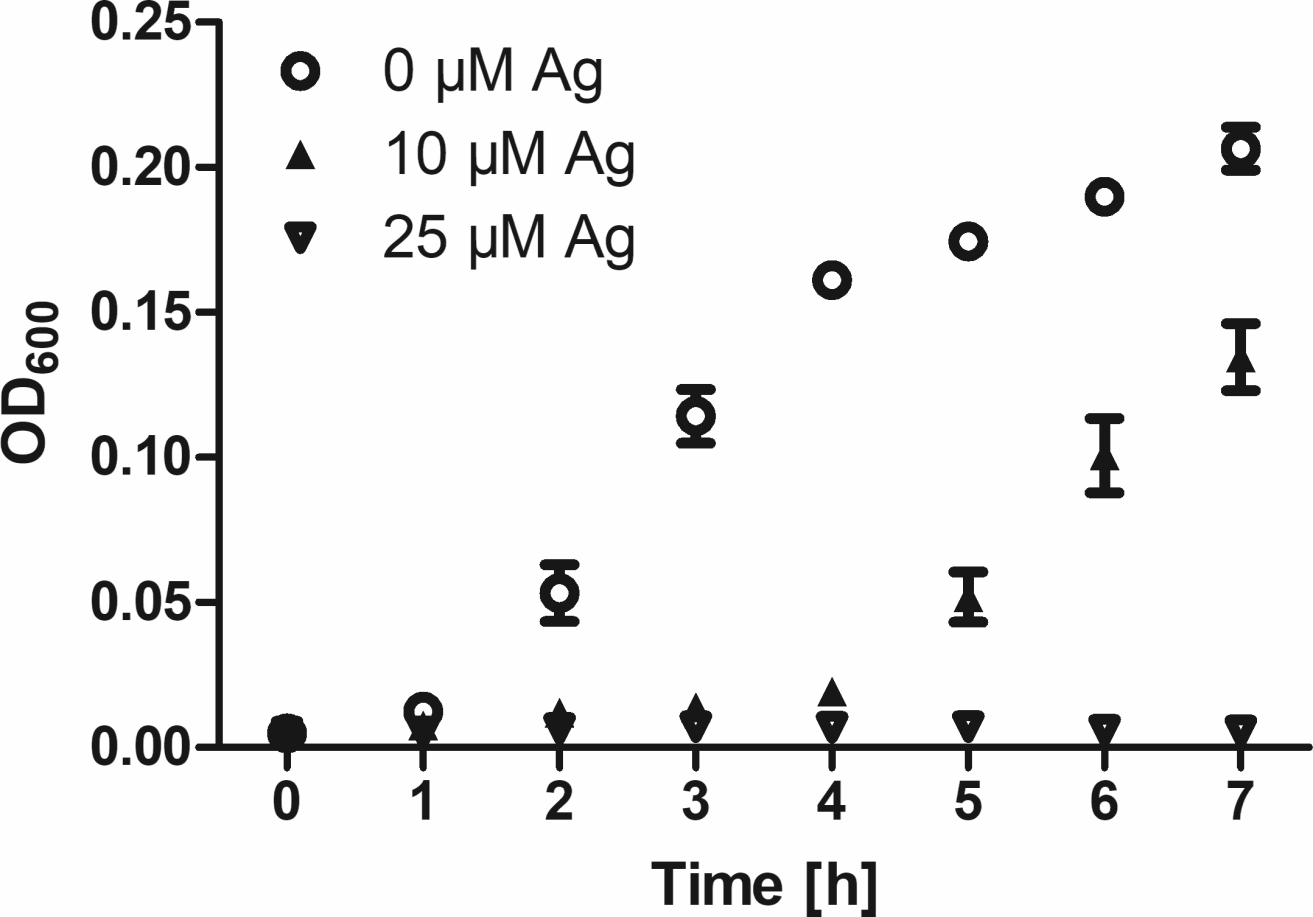
OD_600_ assay at different time points. Viability of *E*. *coli* cultured in the presence of the indicated concentrations of AgNO_3_. Data are shown as means +S.E.M. of n = 3 independent experiments.

Determination of the OD_600_ confirms, in principle, the results of the MTT assay for silver-furnished textiles (Fig 3). In contrast, no loss of viability is observed for copper-containing coatings. Notably, copper release leads to discoloration of the culture media. Measuring the absorption of media incubated with copper-treated textile in the absence of bacteria indicates a significant absorption at 600 nm. If these values are subtracted as background, a reduction of viability is observed for copper as well (Fig 4). As the OD_600_ is a non-destructive assay, it allows for time-resolved measurements instead of a single endpoint measurement. As shown in Fig 5, culture in the presence of 10 and 25 μM Ag^+^ both inhibit the growth of *E*. *coli* during the standard assay duration of 3h. However, at 10 μM Ag^+^ bacterial growth is only delayed by 3 hours, as shown by their entry into the phase of exponential growth 4h after the start of the experiment.

**Fig 6:**
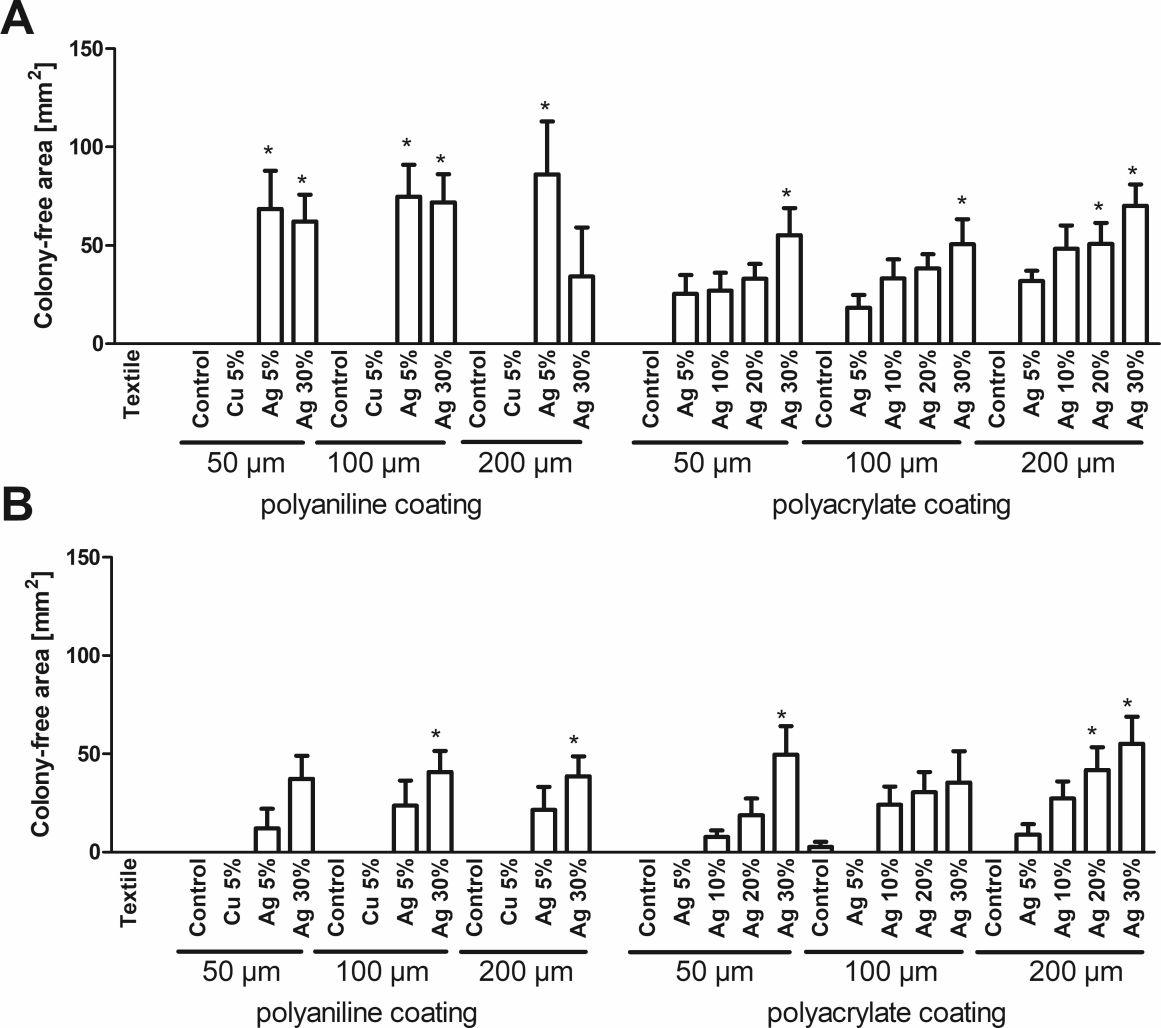
Disk diffusion assay. The area of the bacteria-free zone around textile disks placed on agar plates coated with *E*. *coli* (A) or *S*. *warneri* (B) was measured. Data are shown as means +S.E.M. of n = 4 independent experiments. *Mean significantly different from untreated textile; One-way ANOVA with Tukey´s post-hoc test (p < 0.05).

In the disk diffusion assay (Fig 6), higher values indicate antibacterial efficiency, as they represent the area of growth inhibition around the textile sample on an agar plate. Uncoated polyester fabric and samples without addition of silver or copper pigments to the coating are ineffective. Notably, all copper-containing coatings do not show any bactericidal activity, as well. In contrast, silver-containing coatings abrogate bacterial growth in the vicinity of the textile samples. In general, these effects correlate with the amount of silver pigment added to the coating, except for the case of polyaniline-coated samples applied to *E*. *coli*.

**Fig 7:**
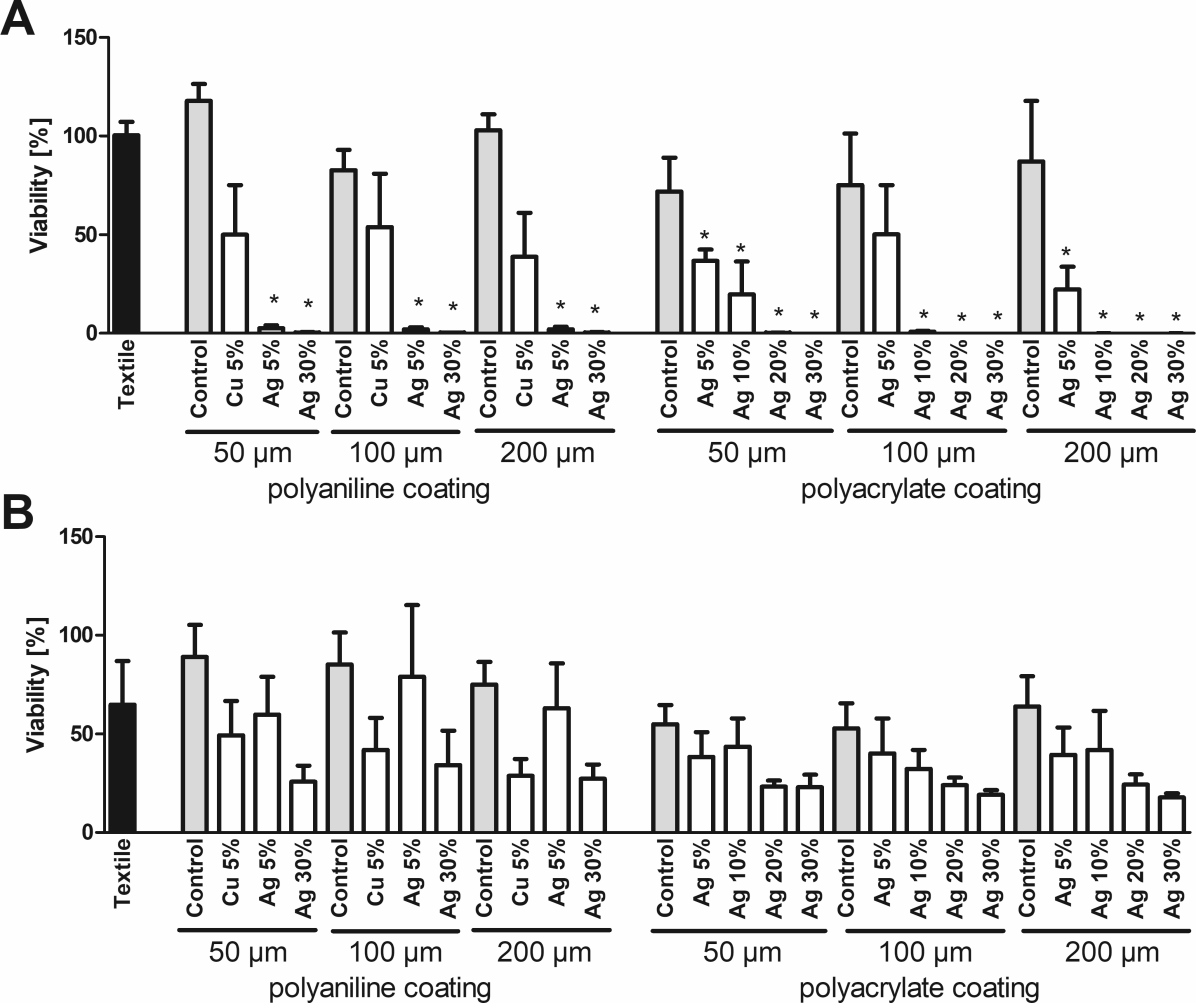
Colony^(Sol)^ assay. Viability of *E*. *coli* (A) or *S*. *warneri* (B) after 3h culture in the presence of textile samples was analyzed by colony formation. A viability of 100% corresponds to the signal obtained with bacteria in the absence of any textile samples. Data are shown as means +S.E.M. of n = 3 independent experiments. *Mean significantly different from untreated textile; One-way ANOVA with Tukey´s post-hoc test (p < 0.05).

In the next experiment, bacteria are cultured in the presence of textile samples as for MTT and OD_600_ assays, followed by counting the numbers of colonies after plating diluted aliquots onto agar plates (Fig 7). The effects on bacterial viability correspond well with the results from both other methods, except for a somewhat higher toxicity of silver-coated textiles on *E*. *coli*. Notably, this method involving serial dilution allows for a more precise determination of low numbers of viable bacteria. When the *E*. *coli* data from Fig 7, which show no apparent remaining viability for many silver-containing samples, are depicted on a logarithmic scale, this allows the sensitive quantification of low bacterial numbers over several log steps (S3 Fig).

In the final set of experiments, textile samples are soaked in bacterial solution, bacteria recovered by elution with sodium chloride solution and diluted in their respective culture media, followed by determining the remaining bacterial viability by colony formation as in the previous experiment. The resulting viability is shown as the percentage of colonies recovered after 3h incubation, compared to the immediate recovery directly after soaking. While *E*. *coli* gives results that are in good agreement with the other methods, no notable differences between metal-free controls and silver-containing polyacrylate coatings are observed for *S*. *warneri*.

To compare the results of the different methods, the Pearson product-moment correlation coefficient was determined (Table 2). For *E*. *coli*, statistically significant linear correlations (p<0.05) were observed between all methods. However, the correlation coefficients indicate that not all correlations can be considered to be strong, indicating a certain degree of variation between the different tests. The negative correlation coefficients for disk diffusion are not an indication of poor correlation, but result from the fact that in this case data cannot be expressed as percent viability, but are indicated as area without bacterial growth instead, which is inversely proportional to viability. For *S*. *warneri*, the Colony^(Soak)^ assay showed no statistically significant correlation with the disk diffusion assay, which was the only correlation with a p value greater 0.05.

**Table 2:** Correlation analysis.

	MTT	OD_600_	Disk Diffusion	Colony^(Sol)^	Colony^(Soak)^	
**MTT**		0.897	-0.516	0.820	0.640	*E*. *coli*
		(p<0.001)	(p = 0.005)	(p<0.001)	(p<0.001)	
**OD**_**600**_	0.914		-0.873	0.964	0.665	
	(p<0.001)		(p<0.001)	(p<0.001)	(p<0.001)	
**Disk Diffusion**	-0.570	-0.856		-0.833	-0.462	
	(p = 0.002)	(p<0.001)		(p<0.001)	(p = 0.013)	
**Colony**^**(Sol)**^	0.654	0.732	-0.624		0.595	
	(p<0.001)	(p<0.001)	(p<0.001)		(p = 0.001)	
**Colony**^**(Soak)**^	0.524	0.608	-0.196	0.585		
	(p = 0.004)	(p = 0.001)	(p = 0.319)	(p = 0.001)		
	*S*. *warneri*					

Pearson-correlation between the results obtained with the five different toxicity assays, expressed by correlation coefficients and p values. Data points for copper-coated textiles obtained with the OD_600_ method were excluded from the analyses due to interference by leaching from the textiles.

## Discussion

Various protocols are routinely being employed to determine the antibacterial effectiveness of textile treatments [2]. Comparison of the results is hindered by the lack of information regarding the reproducibility and comparability between the different assays. The present study was initiated to provide a first overview on the comparability of some commonly used methods by using one set of samples based on coatings containing copper or silver as the anti-bacterial agent, which are tested in parallel with five different assays.

In general, the results from all five methods are in good agreement, although different endpoints are investigated. Metabolic activity is used for the MTT assay [24]. It will not provide information on the number of viable bacteria, as it cannot distinguish between a reduction resulting from lower numbers of bacteria with unaltered metabolic activity, and equal total numbers of bacteria that have differential metabolic activity. In contrast, the OD_600_ is proportional to the total number of bacteria, but does not provide any information regarding metabolic activity or their viability. For disk diffusion and the methods depending on colony formation, only viable, dividing bacteria are relevant, whereas dead bacteria or even bacteria in a temporary growth arrest will remain undetected.

Several limitations exist for the different methods. Disk diffusion strictly depends on the diffusion and solubility of the antibacterial substance from the textile into the surrounding agar [11]. Even extraordinarily efficient antibacterial agents will show no effect, if they stay bound to the textile or are unable to diffuse in the agar, e.g., due to precipitation. The latter may be the reason why the copper coated textiles, which show considerable toxicity in other assays, have no effect in the disk diffusion assay.

The OD_600_ method allows for a simple time-resolved monitoring of bacterial growth, because bacteria are not lysed and can be measured repeatedly. However, the application for antimicrobial testing is limited to textiles without leaching from coatings with absorption at this wavelength, or necessitates running parallel experiments for background correction, as in Fig 4. Alternatively, light scattering by bacteria is not limited to a particular wavelength. This opens the possibility to perform the assay at any different wavelength, if it is undisturbed by the specific samples. Leaching might also affect the absorption in the MTT assay measured at 570 nm. However, due to the specific absorption peak of MTTs formazan, this assay allows for parallel measurement of a reference wavelength, thereby strongly reducing interference by non-specific absorption.

Silver is known for its strong oligodynamic effect [25], and silver ions have previously been shown to be a far more potent antibacterial agent than copper ions [26], which is confirmed by the measurements in S2 Fig. Yet, this is not observed in the MTT assay or in the assays based on colony formation, where copper seems to be of similar, or in some cases even higher effectiveness than a coating with a comparable amount of silver. However, for both metals, the cationic forms released from the metallic surfaces are known to be the toxic metal species [25,27]. Solubilization of the baser metal, copper, is much higher than for silver, as can be seen in Fig 2. Applied in its metallic form, copper can show higher antibacterial activity than silver [28]. Compared to the toxicity data in S2 Fig, the total concentrations of copper and silver released from the textile coatings (Fig 2) should cause much stronger decreases in viability as the ones observed in the MTT assay (Fig 1). This suggests that for both metals, not only highly toxic ionic species, but also comparatively less toxic metallic species were mobilized from the coatings. These cannot be distinguished by the AAS measurements used to measure metal release into the culture media.

Experiments in the present study were performed in bacterial culture media, which may not ideally resemble the chemical environment of the bacteria in a given application. The impact of the liquid phase on the dissolution of ions needs to be considered. In case of skin contact, this could be done by using synthetic sweat. For copper it has already been shown that the composition is an important factor; the sodium content in synthetic sweat influences the amount of copper dissolution from metallic surfaces [29].

One other relevant factor in choosing a suitable assay is the time required to perform it, especially hands on time. As shown in Table 3, the assays vary considerably for this parameter, from 10 minutes to up to nearly 2h for the sample panel investigated in the present study. Hereby methods involving colony formation are inherently more time consuming than assays based on photometric detection of their respective endpoint. On the one hand, the time required for the colonies to grow necessitates at least overnight incubation. On the other hand, counting colonies, especially if it is performed manually, is very laborious and time consuming. However, the logarithmic dilution yields a far superior dynamic range. The photometric detection of absorption or turbidity is limited by the sensitivity of the instrument and background of the assays. While it permits the quantification of a reduction by up to 95% of the initial number of bacteria, a reliable quantification below this range is not possible. In contrast, colony formation allows determining the number of remaining viable bacteria over several orders of magnitude. Due to the short doubling times, even a few remaining bacteria can expand to a significant population under the right conditions, constituting a potential health hazard. In the medical field, the effectiveness of sanitization, disinfection or even sterilization is measured by the log_10_ reduction of microbes. E.g., sterilization requires at least a reduction by six log_10_ steps [30].

**Table 3:** Time requirement.

Method	Time		
	Hands on	Incubation	Total
**MTT-Assay**	25min	3h 35min	4h
**OD**_**600**_	10min	3h	3h 10min
**Disk Diffusion**	15min	24h	24h 15min
**Colony**^**(Sol)**^	1h 40min	27h	28h 40min
**Colony**^**(Soak)**^	1h 50min	27h	28h 50min

**Fig 8:**
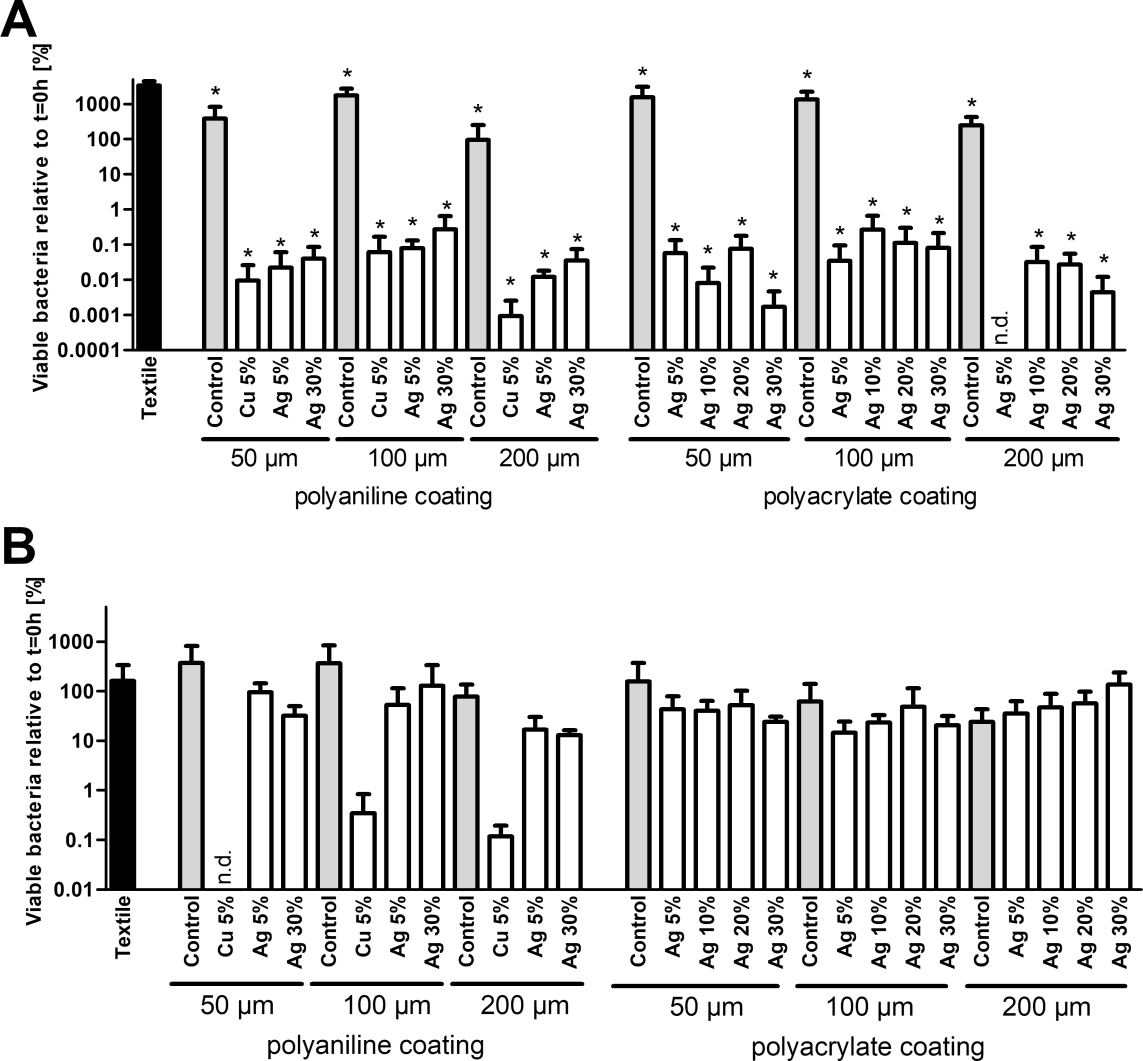
Colony^(Soak)^ assay. Viability of *E*. *coli* (A) or *S*. *warneri* (B) after 3h culture on textile disks was analyzed by colony formation. To illustrate the resolution in the low viability range, values are depicted on a logarithmic scale. Data are shown as means +S.E.M. of n = 3 independent experiments. *Mean significantly different from untreated textile; One-way ANOVA with Tukey´s post-hoc test (p < 0.05). n.d.: not detected.

An assay of antibacterial activity should match the functional principle and intended application of the functionalized textile. For example, a textile treatment that prevents colonization by impairing bacterial adhesion might be highly effective against bacterial colonization of its surface. However, it will seem completely ineffective in the MTT, OD_600_, disk diffusion or Colony^(Sol)^ assays, because these tests exclusively measure bactericidal effects, but not passive antibacterial properties. For several scenarios, e.g., when contaminated material is spilled onto a textile surface, methods involving the recovery of viable bacteria from cloth are in closest resemblance to realistic applications. This is the principle of choice for the method according to the AATCC [17], in which textiles are soaked with a solution containing bacteria. These are later recovered after an incubation period by rinsing with saline, followed by detection based on colony formation. Hypothetically, all other methods (MTT, disk diffusion, OD_600_) in this paper could also be used instead of colony formation for analyzing the amount of recovered bacteria. In the present paper we performed colony formation from recovered bacteria and after incubation in bacterial culture medium in the presence of textile samples. In comparison, the effectiveness of the Colony^(Soak)^ assay (Fig 8) showed hardly any effectiveness against *S*. *warneri* by silver in polyacrylate coating, causing a poor correlation with Colony^(Sol)^ and the other assays. While the reason remains unknown, it might be related to a differential availability of silver ions from the surface, which can have a significant impact on the antibacterial effectiveness of metals [27].

There have been reports about differences between Gram-positive and -negative bacteria in the susceptibility to silver ions. The thicker cell wall of Gram-positive bacteria has been suggested to provide protection against the penetration of silver ions into the cytoplasm, leading to lower toxicity [31]. Such an effect cannot be concluded from the present study, as only one representative species from each group was investigated and a difference between the two types of bacteria was not found with every method. Yet, some differences between the susceptibilities of *E*. *coli* and *S*. *warneri* were observed, e.g., the effect of polyacrylate/silver coatings in Fig 8. Accordingly, in order to yield meaningful results, test organisms should be carefully chosen with respect to their relevance for the intended applications of the textile.

## Conclusions

When performing antibacterial testing of textiles, several important points have to be considered to identify an appropriate test system for the particular type of finishing. While the different methods yielded results that were generally comparable, their suitability depends on the specific analytical requirements. This includes factors such as appropriate endpoints for analyzing passive or active antibacterial effects, a potential interference by leaching, hands on time, and the required sensitivity.

## Supporting information

S1 Fig Growth curves of *E*. *coli* and *S*. *warneri*. 1:250 diluted overnight cultures were incubated in 96-well plates for 7h at 37°C in an orbital shaker rotating at 250 rpm. Every hour the absorption at 600 nm (OD_600_) was determined. Data are shown as means of n = 3 independent experiments (5 replicates each) ± S.E.M.
(PDF)

S2 Fig Impact of Ag and Cu ions on the viability of *E*. *coli* and *S*. *warneri*. Bacteria were incubated in their respective culture media in the presence of the indicated concentrations of AgNO_3_ or CuSO_4_. After 3h, viability was determined by the MTT assay. Data are shown as means ± S.E.M. of n = 3 independent experiments, each performed in 8 replicates. Sigmoidal dose–response curves were fitted by non-linear regression.
(PDF)

S3 Fig Logarithmic representation of the colony formation data from *E*. *coli*. Data for *E*. *coli* from Fig 7A are depicted on a logarithmic scale to illustrate the resolution at low concentrations of remaining bacteria. Data are shown as means +S.E.M. of n = 3 independent experiments. *Mean significantly different from untreated textile; One-way ANOVA with Tukey´s post-hoc test (p < 0.05).
(PDF)
