# Supplementary material for: Comparison of methods for determining the effectiveness of antibacterial functionalized textiles
Source: PLoS One. 2017 Nov 21;12(11):e0188304. doi: 10.1371/journal.pone.0188304 (PMC5697868; doi:10.1371/journal.pone.0188304)
Supplement: S1 Fig — 1:250 diluted overnight cultures were incubated in 96-well plates for 7h at 37°C in an orbital shaker rotating at 250 rpm. Every hour the absorption at 600 nm (OD600) was determined. Data are shown as means of n = 3 independent experiments (5 replicates each) ± S.E.M. (PDF) [file pone.0188304.s001.pdf]

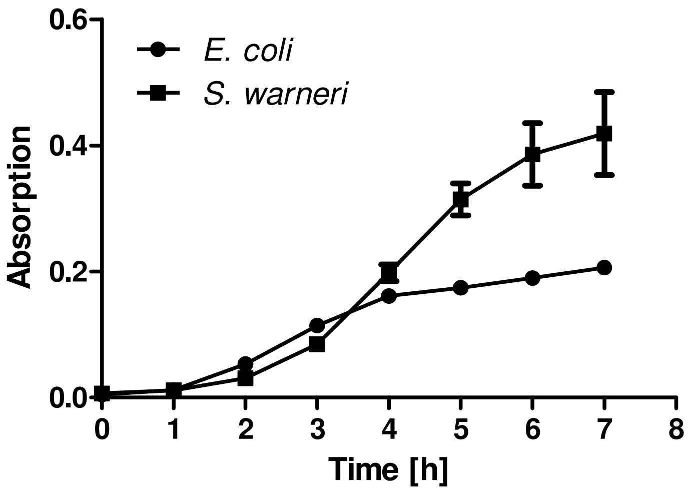

S1 Fig. Growth curves of *E. coli* and *S. warneri*.

1:250 diluted overnight cultures were incubated in 96-well plates for 7h at 37°C in an orbital shaker rotating at 250 rpm. Every hour the absorption at 600 nm (OD_{600}) was determined. Data are shown as means of n=3 independent experiments (5 replicates each) ± S.E.M.
