# Supplementary material for: Comparison of methods for determining the effectiveness of antibacterial functionalized textiles
Source: PLoS One. 2017 Nov 21;12(11):e0188304. doi: 10.1371/journal.pone.0188304 (PMC5697868; doi:10.1371/journal.pone.0188304)
Supplement: S2 Fig — Bacteria were incubated in their respective culture media in the presence of the indicated concentrations of AgNO3 or CuSO4. After 3h, viability was determined by the MTT assay. Data are shown as means ± S.E.M. of n = 3 independent experiments, each performed in 8 replicates. Sigmoidal dose–response curves were fitted by non-linear regression. (PDF) [file pone.0188304.s002.pdf]

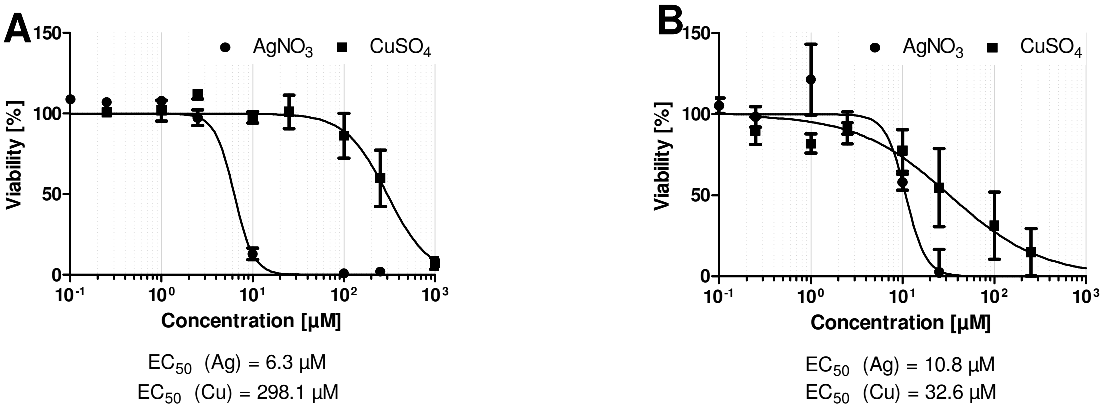

S2 Fig. Impact of Ag and Cu ions on the viability of *E. coli* and *S. warneri*.

Bacteria were incubated in their respective culture media in the presence of the indicated concentrations of $AgNO_3$ or $CuSO_4$. After 3h, viability was determined by the MTT assay. Data are shown as means ± S.E.M. of n=3 independent experiments, each performed in 8 replicates. Sigmoidal dose–response curves were fitted by non-linear regression.
