# Supplementary material for: Comparison of methods for determining the effectiveness of antibacterial functionalized textiles
Source: PLoS One. 2017 Nov 21;12(11):e0188304. doi: 10.1371/journal.pone.0188304 (PMC5697868; doi:10.1371/journal.pone.0188304)
Supplement: S3 Fig — Data for E. coli from Fig 7A are depicted on a logarithmic scale to illustrate the resolution at low concentrations of remaining bacteria. Data are shown as means +S.E.M. of n = 3 independent experiments. *Mean significantly different from untreated textile; One-way ANOVA with Tukey´s post-hoc test (p < 0.05). (PDF) [file pone.0188304.s003.pdf]

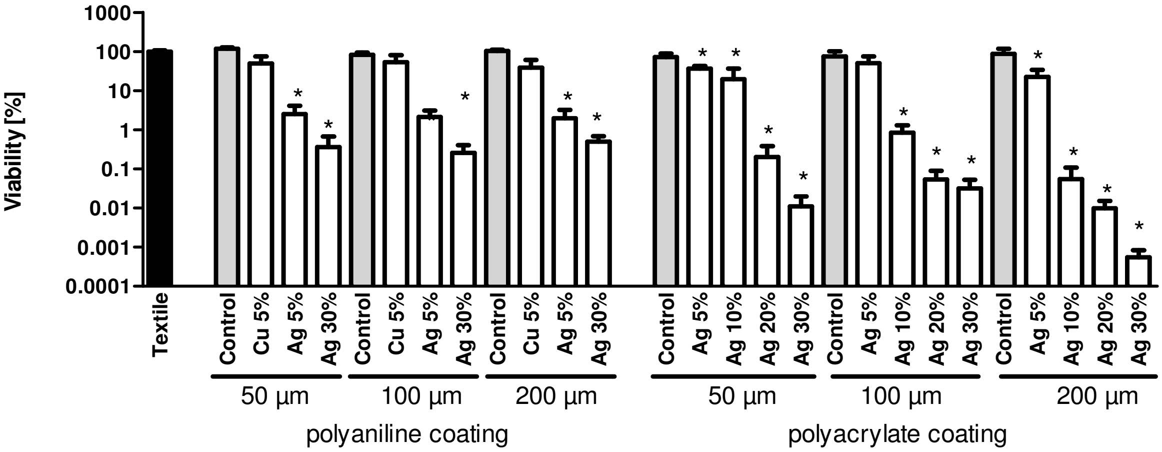

S3 Fig. Logarithmic representation of the colony formation data from *E. coli.*

Data for *E. coli* from Fig. 7A are depicted on a logarithmic scale to illustrate the resolution at low concentrations of remaining bacteria. Data are shown as means +S.E.M. of n=3 independent experiments. *Mean significantly different from untreated textile; One-way ANOVA with Tukey´s post-hoc test ($p < 0.05$).
